# Supplementary material for: A Critical Review of the Pharmacokinetics, Pharmacodynamics, and Safety Data of Antibiotics in Avian Species
Source: Antibiotics (Basel). 2022 May 31;11(6):741. doi: 10.3390/antibiotics11060741 (PMC9219738; doi:10.3390/antibiotics11060741)
Supplement: Supplementary file 1 [file antibiotics-11-00741-s001.zip › antibiotics-1692539-supplementary.pdf]

Table S1. Characteristics of included studies.

Study	Species (English name)	Species (scientific name)	Dosing regimen	Sample size (n)	Condition of birds	Time points for data collection	Control	Environment	Comments
**Enrofloxacin**									
[48]	Caribbean flamingo	Phoenicopterus ruber ruber	PO 15mg/kg SD	7	Healthy	0.25, 0.5, 1, 2,4,8,12, and 24h post-administration	None	Have access to indoor facility & outdoor exhibit	-
			SC 15mg/kg SD	7	Healthy				
[45]	Southern crested caracara	Caracara plancus	IV 5 mg/kg SD	4	Healthy	0,5,15,35min and 1,2,4,6,8,10,12,24,29,34h after injection	None	-	-
			IM 10mg/kg SD	4	Healthy	0,10,20,40min and 1,2,4,6,8,10,12,24,29,34h after injection	None		
[42]	African penguin	Spheniscus demersus	IV 15mg/kg SD	5	Healthy	0, 0.5, 1,2,4,8,12,24,36, and 48h	None	24 °C	-
			PO pill 15mg/kg	5	Healthy		None		
			PO pill in fish 15mg/kg	5	Healthy		None		
[35]	Common ostrich	Struthio camelus	IM 15mg/kg q12h x 3 days	6	Healthy	Before and at 10,20,30,45 min and at 1,1.5,2,3,4,6,8,12 and	None	-	-

			SC 15mg/kg q12h x 3 days	6	Healthy	24h after the first administration. Before and 2h after each subsequent administration.	None		
[44]			IV 5mg/kg SD	5	Healthy	0,5, 10,15,30, 45,60, 90 min and 2,3,4,6,8,10,12, 24,48 and 72h after dosing	None	-	-
			IM 5mg/kg SD	5	Healthy		None		
[46]	Emu	Dromaius novaehollandiae	IV 2.2 ± 0.03 mg/kg SD	3	kyphosis, lordosis and scoliosis since young but otherwise healthy	7.5, 15, 30, 60, 90 min and 2,4,6,8, 12,16 and 24h post-injection	None	Kept indoors	-
[34]	Greater rhea	Rhea americana albescens	IM 15mg/kg SD	6	Healthy	0,5,10,15,30,45,60,90 mins and at 2,3,4,6,8,10, 24,48h after dosing	None	-	-
[4]	Houbara bustard	Chlamydotis undulata macqueenii	IM 10mg/kg SD	8	Healthy	0.25, 0.50, 0.75, 1, 2, 3, 4, 6, 8, 10, 12, 24,48h	None	19.8 - 45.7°C, 13.3-13.5h of light	-
			PO 10mg/kg SD	8	Healthy		None		
			IV 10mg/kg SD	8	Healthy	0.25, 0.50, 0.75, 1, 2, 3, 4, 6, 8, 10, 12, 24,48h and at 5min	None	26.5 – 46.7°C, 12.8h of light	

[37]	Red-tailed hawk	Buteo jamaicensis	PO in prey 15mg/kg SD	8	Chronic muco-skeletal or ocular abnormalities present but otherwise healthy	0.25, 0.5, 1, 2, 4, 8, 12, 24, 48h after administration	None	-	Fasted 12h before and 13h after drug administration
			IM 15mg/kg SD	8			None		
			IV 15mg/kg SD	8			None		
	Great horned owl	Bubo virginianus	PO in prey 15mg/kg SD	5			None		
			IM 15mg/kg SD	5			None		
			IV 15mg/kg SD	5			None		
[36]	African grey parrot	Psittacus erithacus	IM 15mg/kg SD	12	Healthy	0.5, 1,2,4,6,8,12,24h after administration	None	Indoors, controlled temperature and lighting	-
			PO 3mg/kg SD	12	Healthy		None		
			PO 15mg/kg SD	12	Healthy		None		
			PO 30mg/kg SD	12	Healthy		None		
			PO 30mg/kg q12 x 10 days	4	Healthy	6,8,12h following administration during the first dosage interval of day 1 and the last dosage interval on day 10	4 birds received PO physiological saline		
[47]	Greater rhea	Rhea americana albescens	IV 15mg/kg SD	6	Healthy	0,5,10,15,30,45,60,90 mins and at 2,3,4,6,8,10,24,48h after dosing	None	-	-

[49]	African grey parrot	Psittacus erithacus	0.09, 0.19, 0.38, 0.75, 1.5 and 3.0 mg/mL x 7 days	4 birds each	Healthy	8 am and 3pm on treatment days 4 and 7	4 birds received unmedicated water	13h light (7am-8pm)	-
	Common pheasant	Phasianus colchicus L.	IV 10mg/kg SD	6	Healthy	8:00-8:30 AM at 0.25, 0.5, 1,2,4,6,8,10,24h after drug administration	None	-	-
			PO 10mg/kg SD	6					Fasted 12h prior to drug administration
**Marbofloxacin**									
[50]	Eurasian buzzard	Buteo buteo	IO 2mg/kg SD	5	Healthy	0,5,15,30,45,60min and 2,4,6,8,10,12,24h	None	-	-
[51]	Eurasian griffon vulture	Gyps fulvus	IV 2mg/kg SD	6	Healthy		None	-	-
[52]	Blue and gold macaw	Ara ararauna	IV 2.5mg/kg SD	10	Healthy	0, 0.5, 1,3,6,12,24,48,72,96h after administration	None	Housed outdoors	-
			PO 2.5mg/kg SD	10	Healthy		None		
[53]	Common ostrich	Struthio camelus	IV 5mg/kg SD	6	Healthy	0,5,10,15,30,45,60,90 min and 2,3,4,6,8,10,12,24,48,72h after dosing	None	-	-
			IM 5mg/kg SD	6	Healthy		None		
	Common pheasant	Phasianus colchicus L.	IV 5mg/kg SD	6	Healthy	8:00-8:30 AM at 0.25, 0.5, 1,2,4,6,8,10,24h after drug administration	-	-	-
			PO 5mg/kg SD	6					Fasted 12h prior to drug

									administration
[54]	Eurasian buzzard	Buteo buteo	IV 2mg/kg SD	4	Healthy	0,5,15,30,45,60min and 2,4,6,8,10,12,24,30h post-administration	None	-	-
[56]	Eurasian buzzard	Buteo buteo	PO 10mg/kg SD	8	Healthy	0, 0.25, 0.5, 0.75, 1,2,4,8,6,12,24,36h,48h post-administration	None	Outdoor flight enclosure	Fasted 24h before start of study
**Gentamicin**									
[57]	Budgerigar	Melopsittacus undulatus	IM 5mg/kg SD	20	Healthy	15,30,45,60,120minutes after drug administration	-	-	-
			IM 10mg/kg SD	20	Healthy				
[62]	Scarlet macaw	Ara macao	IM 5mg/kg SD	30	Healthy	0.5,1,3,6,9, and 12h after drug administration	None	-	-
			IM 10mg/kg SD	30	Healthy		None		
			IM 5mg/kg q12h x 7 days	10	Healthy	0.5 and 12h after drug administration	5 birds given IM physiological saline		
	Galah	Eolophus roseicapilla	IM 5mg/kg SD	15	Healthy	0.5,1,3,6,9, and 12h after drug administration	None		
			IM 10mg/kg SD	15	Healthy		None		
			IM 5mg/kg q12h x 7 days	10	Healthy	3 and 12h after drug administration	5 birds given IM		

							physiological saline		
[58]	Great horned owl	Bubo virginianus	IM 10mg/kg q12h x 5 days	8	Healthy		8 owls given sterile saline	-	-
[59]	Red-tailed hawk	Buteo jamaicensis	IV 10mg/kg q12h x 4 days	5	Permanently crippled but otherwise healthy	Days 1,2, and 4	None	-	-
			IV 20mg/kg q12h x 6 days	5			None	-	-
[60]	Lanner falcon	Falco biarmicus	IM 5mg/kg OD x 4 days	2	Severe respiratory tract infection with rapid weight loss	Not mentioned	none	-	-
**Amikacin**									
[61]	Cockatiel	Nymphicus hollandicus	IM 5mg/kg q12h x 3 days	12	Healthy	before the sixth injection and at 1.5, 3.3, 4.5, 6 and 7h following the sixth injection	2 birds given IM lactated ringers solution	Outdoor aviary	-
	Ring-necked pheasant	Phasianus colchicus	IM 5mg/kg SD	5	Healthy		None		
			IM 10mg/kg SD	7	Healthy		None		

			IM 20mg/kg SD	3	Healthy		None		
	Greater sandhill crane	Grus canadensis tabida	IM 5mg/kg SD	8	Healthy		None		
			IM 10mg/kg SD	1	Healthy		None		
			IM 20mg/kg SD	1	Healthy		None		
[64]	Red-tailed hawks	Buteo jamaicensis	IV 10mg/kg SD	5	Wing injuries but otherwise healthy	0, 0.5, 1,2,4,6,8,10,18 and 24h post injection	None	21-23°C, 10h light	-
			IM 10mg/kg SD	5			None		
	Great horned owl	Bubo virginianus	IV 10mg/kg SD	5			None		
			IM 10mg/kg SD	5			None		
	Golden eagle	Aquila chrysaetos	IV 10mg/kg SD	3			None		
			IM 10mg/kg SD	3			None		
[68]	Blue-fronted amazon parrot	Amazona aestiva aestiva	IV 15mg/kg SD	8	Healthy	immediately, 15, 30, and 1,2,4,6,8h after IV administration	None	26-30 °C, 12h light	-
			IM 15mg/kg SD	8	Healthy	1,2,4,6,8h after IM administration	None		
[69]	Red-tailed hawks	Buteo jamaicensis	IM 20mg/kg SD	6	Healthy	5,15,30,45min and 1,3,4,6,8,10,12, and 24h post-injection	None	-	-

[70]	Emu	Dromaius novaehollandiae	IV 7.2 ± 0.12 mg/kg SD	3	Healthy	5,15,30,45,60,75,90 min and 2,4,5,6,8,10,12 and 24h post-injection	None	Indoor enclosure	-
[67]	African grey parrot	Psittacus erithacus	IV 5mg/kg SD	30	Healthy	0,5,15,30 and 45min and 1,2,4,8,24h post-administration. 0min is before injection	None	-	-
			IV 10mg/kg SD	30	Healthy		None		
			IV 20mg/kg SD	30	Healthy		None		
			IM 5mg/kg SD	24	Healthy	0,15,45min and 1,2,4,8,24h post-administration	None		
			IM 10mg/kg SD	24	Healthy		None		
			IM 20mg/kg SD	24	Healthy		None		
**Ceftiofur**									
[73]	Red-tailed hawks	Buteo jamaicensis	IM 10mg/kg SD	7	Healthy	-	None	-	-
			IM 20mg/kg SD	7	Healthy		None		
[74]	Ringneck dove	Streptopelia risoria	IM 50mg/kg SD	30	Healthy	0, 0.5, 1,2,4,8,12,24,48,72,96,120,144, 168 and 192h post injection	6 untreated birds	-	-
[75]	Cattle egret	Bubulcus ibis	IM 20mg/kg SD	18	Healthy	1,2,4,8,12,24,48,72,96,120,144, 168,192,216,240h	None	-	-
[72]	Cockatiel	Nymphicus hollandicus	IM 10mg/kg SD	60	Healthy	0, 0.5, 1, 2,4, 8,12h	None	-	-
[72]	Orange-winged	Amazona amazonica	IM 10mg/kg SD	27	Healthy	0.5, 1,2 ,4 12, 24h	None		

	amazon parrot								
[77]	Helmeted guineafowl	Numida meleagris	IM 10mg/kg SD	14	Healthy	-	None	-	-
[76]	American flamingo	Phoenicopterus ruber	IM 10mg/kg SD	11	Healthy	just prior to administration and 2,4,8,12,24,32,48,56,72,96,144 and 192h post administration	None	-	-
			SC 10mg/kg SD	2	Healthy		None		
[78]	American black duck	Anas rubripes	IM 10mg/kg SD	14	-	-	None	-	-
**Doxycycline**									
[79]	Houbara bustard	Chlamydotis undulata	IM 100mg/kg x 7 doses at intervals of 7,7,7,6,6,5 days	10	Healthy	After the first and seventh injection at the following intervals: 0 (before injection),12,24,36,48,72,96,144,168 (just before the second injection),180h	None	9.4-31.8 °C, 13h light	-
			SC 100mg/kg x 7 doses at intervals of 7,7,7,6,6,5 days	4	Healthy		None		
[80]	Orange-winged amazon parrot	Amazona amazonica	IM 100mg/kg commercial formulation (20mg/mL) SD	5	All were slightly to moderately overweight at the start but were	3,24,48,72,96,120,144 hours after injection 22,23,26	None	18-22°C, 12h light	-
			IM 100mg/kg PC 75mg/mL SD	4		3,12,24,32,48,72,96,168 hours after injection	None		

			IM 100mg/kg (PC 100mg/mL) SD	4	otherwise healthy	0900-1030 on days 1,3,7,10,13,15,17,20 after injection	None		
	Goffin's cockatoo	Cacatua goffini	IM 100mg/kg (PC 75mg/mL) SD	4	Healthy	3,12,24,32,48,72,96,168 hours after injection	None		
			IM 100mg/kg (PC 100mg/mL) SD	4	Healthy	0900-1030 on days 1,3,7,10,13,15,17,20 after injection	None		
	Tinneh African grey parrot	Psittacus Erithacus tinneh	IM 100mg/kg (PC 100mg/ml) SD	4	Healthy	0900-1030 on days 1,3,7,10,13,15,17,20 after injection	None		
			SC 100mg/kg (PC 100mg/mL) SD	4	Healthy		None		
[81]	Cockatiel	Nymphicus hollandicus	IM 100mg/kg q10 days x 5 doses	6	Healthy	7am on days 7,10,20,30,40,51	4 birds fed unmedicated pellets and water	18-25°C, 12h light	No calcium or other dietary supplements were provided
			0.28mg/mL x 45 days MW	3	Healthy	7am on days 10,20,30,45			
			0.83mg/mL x 45 days MW	6	Healthy	7am on days 3,7,15,25,35,45			
			500mg/kg seed mixture x 45 days	6	Healthy		6 birds fed unmedicated mixed		

							seeds and water		
			1000mg/kg mash x 45 days	6	Healthy	7am on days 3 and 7	6 birds fed unmedicated mash and water		
[82]	Cockatiel	Nymphicus hollandicus	300mg/kg of pellets x 47 days	9	Healthy	830am on days 3,7,14,21,28,35,42	6 birds fed unmedicated pellets	20-24°C, 10h light	No cuttlebone, mineral blocks or other nutritional supplements provided
[83]	Cockatiel	Nymphicus hollandicus	400mg/L x 30 days MW	11	Naturally infected with spiral bacteria	-	7 birds naturally infected with spiral bacteria fed unmedicated water	-	-
[84]	Budgerigar	Melopsittacus undulatus	300mg/kg of seeds x 42 days	-	Healthy	morning on treatment days 4,7,14,21,28,35,42 or in the	Fed unmedica	-	-

			0,50,100,200,400mg/L x14d MW	-	Healthy	afternoon on days 12,26, and 40	ted seeds and water		
[85]	African grey parrot	Psittacus Erithacus tinneh	400mg/L x 7 days MW	4	Healthy	Days 3 and 7	None	18-22°C, 12h light	Birds switched from pelleted feed to commercial parrot seed diet 8 days before starting the trial to avoid possible chelation of doxycycline by calcium present in the pelleted feed.
			800mg/L x 7 days MW	4	Healthy		None		
			800mg/L x 42 days MW	7	Healthy	0830 on days 4,7,14,21,28,35,42	4 birds fed unmedicated water		
	Goffin's cockatoo	Cacatua goffini	400mg/L x 7 days	4	Healthy	Days 3 and 7	None		
			800mg/L x 7 days	4	Healthy		None		
			800mg/L x 42 days MW	8	Healthy	0830 on days 4,7,14,21,28,35,42	4 birds fed unmedicated water		
	Orange-winged amazon parrot	Amazona amazonica	400mg/L x 7 days	4	Some were moderately obese but otherwise healthy	Days 3 and 7	None		
			800mg/L x 7 days	4		Days 3 and 7	None		
[86]	Blue-and-gold macaw,	Ara ararauna<br>Ara macao	1000mg/kg of corn x 45 days	10<br>3	Healthy	Days 3,15,30,45	None	-	-

	scarlet macaw								
[87]	beautiful fruit dove, black-naped fruit dove, Jambu fruit doves, ring-necked dove	Ptilinopus pulchellus, Ptilinopus melanospila, ptilinopus jambu, streptopelia capicola	500mg/L x 45 days MW	18	5/18 birds were tested positive for C.psittaci by PCR testing of choano-cloacal swabs	1100 on days 3,8,14,21,35,42	None	10.5h light	-
[88]	Common ostrich	Struthio camelus	IV 15mg/kg SD	10	Healthy	At 0 (pretreatment), 0.08, 0.25, 0.5,1,2,4,6,8,12,24,48h after administering the drug	None	Isolated open system pen	Food withheld 12h before and 6h after drug administration
			IM 15mg/kg SD	10					
			PO 15mg/kg SD	10					
[89]	Cockatiel	Nymphicus hollandicus	PO crop gavage 35mg/kg q24h x 21 days	8	Healthy cockatiels artificially infected with Chlamydia psittaci	On days 14 and 21 2-4h after drug administration	6 artificially infected and non-treated birds	12h photoperiod	-
			PO crop gavage 35mg/kg q24h x 45 days	8					

[90]	Exotic columbiformes for a zoo (species not mentioned)	-	PO oral gavage 50mg/kg x OD 45d	38	Birds infected with chlamydia psittaci	Not measured	None	-	-
